# Plague and the Mongol conquest of Baghdad (1258)? A reevaluation of the sources

**DOI:** 10.1017/mdh.2023.38

**Published:** 2024-10

**Authors:** Jonathan Brack, Michal Biran, Reuven Amitai

**Affiliations:** 1 Dept. of History, Northwestern University, and Dept. of Middle East Studies, Ben-Gurion University of the Negev; 2 Institute of Asian and African Studies, Hebrew University of Jerusalem; 3 Institute of Asian and African Studies, Hebrew University of Jerusalem, and Ewha Womans University, Seoul

**Keywords:** Black Death, Plague, Mongols, Baghdad, Middle Eastern history, Mamluk historiography, Ilkhanate

## Abstract

This paper reexamines the sources used by N. Fancy and M.H. Green in “Plague and the Fall of Baghdad (1258)” (Medical History, 65/2 (2021), 157–177). Fancy and Green argued that the Arabic and Persian descriptions of the Mongol sieges in Iran and Iraq, and in particular, in the conquest of Baghdad in 1258, indicate that the besieged fortresses and cities were struck by Plague after the Mongol sieges were lifted. This, they suggested, is part of a recurrent pattern of the outbreak of Plague transmitted by the Mongol expansion across Eurasia. Fancy and Green concluded that the primary sources substantiate the theory driven by recent paleogenetic studies indicating that the Mongol conquests of the thirteenth century set the stage for the massive pandemic of the mid-fourteenth century. The link between the Plague outbreak and the Mongol siege of Baghdad relies on three near-contemporaneous historical accounts. However, our re-examination of the sources shows that the main text (in Persian) has been significantly misunderstood, and that the two other texts (in Syriac and Arabic) have been mis-contextualized, and thus not understood properly. They do not support the authors’ claim regarding Plague epidemic in Baghdad in 1258, nor do other contemporary and later Arabic texts from Syria and Egypt adduced by them, which we re-examine in detail here. We conclude that there is no evidence for the appearance of Plague during or immediately after the Mongol conquests in the Middle East, certainly not for its transmission by the Mongols.

## Introduction

One of the significant developments in historical research during the last generation or so has been the increasing cooperation of textual historians with natural scientists and scientifically informed historians in creating a richer and more nuanced picture of the human past. We have seen important advances based on insights and data from climatology, seismology, genetics, medicine, animal husbandry, various aspects of scientific archeology, and surely other fields. Further developments ignite the imagination and all of us look forward to hearing about new research and wonder how it might impact our own fields.

At the same time, the need for careful and exact textual research in original languages – ancient and modern – has not been alleviated. One needs to maintain high standards of philological work, with texts put into proper historical and cultural contexts and the rules of evidence assiduously followed. Only then can a happy marriage between textual historians and those working in the gamut of natural sciences be achieved and sustained.

Recently, two new articles argued for the central role that the Mongols’ conquests in the eastern Islamic world during the thirteenth century played in the dissemination of plague causing the pandemic wave referred to as the ‘Black Death’. According to this recent theory, plague was transmitted from its long-term reservoir of the Tian Shan mountains, in present-day Kyrgyzstan, across Central Asia to Europe and the Middle East through Iran and Iraq (not through the northern steppe), and the disease’s westward transmission was facilitated by the Mongol Empire in the thirteenth century, not the fourteenth century.

This thesis was broached by Monica H. Green in her paper ‘The Four Black Deaths’, and then by Nahyan Fancy and Monica H. Green in ‘Plague and the Fall of Baghdad (1258)’.^1^ The authors presented textual evidence from contemporaneous Persian and Arabic sources from Mongol-ruled Iran and Iraq, and from thirteenth to fifteenth century Arabic chronicles from the Mamluk Sultanate of Syria and Egypt, to support *three main arguments*: first, the Mongol conquests, and especially, the conquest of Baghdad by the forces of Hülegü Khan (grandson of Chinggis Khan, who ruled in the Middle East ca. 1255–1265) in 1258, also entailed the outbreak of plague, which occurred shortly after Mongol sieges were lifted; second, a ‘plague-like disease’, which spread in Syria and Egypt after 1258, was linked to this occurrence of plague during the Mongol conquest of Baghdad. Green and Fancy suggest that additional support for these two arguments is found in a discernable shift in descriptions of plague symptoms by Muslim physicians and religious authors in the second half of the thirteenth century, that is, after the fall of Baghdad to the Mongols.^2^ Their third argument is that this connection between the outbreak in Baghdad and the epidemic in Syria and Egypt, which near-contemporaneous sources made, ‘fell out’ of Arabic histories from the mid-fourteenth century, when a new wave of plague swept the Middle East, Asia, and Europe.

However, a recent article published in the journal *Nature*, on the basis of new aDNA evidence retrieved from three specimens from the Christian (East Syriac) cemetery at Kara-Djigach in modern-day northern Kyrgyzstan, calls into question the hypothesis of the thirteenth-century spread of plague associated with the early Mongol campaigns. The victims of a lethal ‘pestilence’ that struck the area’s denizens in the years 1338–9 were buried in this cemetery in Kara-Djigach.^3^ Green had suggested that the genetic diversification (i.e. the ‘Great Polytomy’) that produced four new branches of the bacterium *Yersinia pestis*, one of which caused the fourteenth century pandemic (the ‘Black Death’), can be dated to the thirteenth century, and thus, to the period of Mongol expansion. Yet, the authors of this recent article have decisively demonstrated that the genetic evidence points to a fourteenth-century emergence, which fits the 1338–9 outbreak near lake Issyk-Kul. Importantly, the authors establish the phylogenetic position of the Kara-Djigach genomes, falling on a node shortly preceding and giving birth to the ‘Great Polytomy’, which is the genetic diversification that created the four new branches of *Yersinia pestis.* They hypothesize that Central Asia trade networks, rather than military campaigns, served to disseminate the bacterium, which re-emerged in the outbreak that occurred in the Black Sea region in 1346, shortly after the 1338–9 plague outbreak near lake Issyk-Kul.^4^

In our article here, we take a different approach to this debate by focusing entirely on the historical evidence used by Fancy and Green. We argue that overall, the textual historical evidence used by Fancy and Green does *not* support their conclusions. First, we examine the authors’ argument about plague outbreak after the Mongol siege of Baghdad. Their thesis relies almost entirely on three near-contemporaneous historical accounts of the siege. However, a re-examination of the sources has led us to conclude that the main text (in Persian) has been significantly misunderstood, and the two other accounts (in Syriac and Arabic) have been mis-contextualized, and thus not understood properly. They do not support the authors’ claim regarding the plague outbreak in Baghdad in 1258. Secondly, we re-examine the sixteen Arabic sources, almost all from Ayyubid and Mamluk Syria and Egypt, that Green and Fancy organized into ‘generations’, and then surveyed to support their arguments that plague spread in the area in the aftermath of the Mongol conquests, and, that ‘the role of epidemic disease in the Mongol attacks was commonly known among chroniclers in Syria and Egypt’.^5^ We can already note that their division of these sources into ‘generations’ is problematic, and instead, we identify four original accounts (*Urtexte*, so to speak) that were copied, summarized, and sometimes also slightly altered by subsequent authors over many decades and even centuries.

We examine these Arabic accounts in detail and reach four main conclusions: first, while the Ayyubid and Mamluk sources describe, indeed, the spread of an epidemic disease (or perhaps diseases) in Syria and Egypt in 1258, the symptoms they describe are not specific to plague, and thus, cannot be used to support Fancy and Green’s argument. Secondly, while the Arabic/Persian term *ṭāʿūn*, which Islamic medical texts associate with plague (characterized by swellings (buboes) and fevers, i.e. Bubonic Plague)^6^ is indeed employed by several Mamluk accounts for this epidemic in 1258, these references can all be traced back to one single, contemporaneous account (by Ibn Wāṣil), on which subsequent authors relied and from which they copied (generally shortening it in the process). This contradicts Fancy and Green’s conclusion that there is an ‘extensive body of evidence in Arabic historical chronicles attesting to the presence of a plague-like disease’.^7^

Thirdly, we find that while some Ayyubid and Mamluk historians did report the outbreak of an epidemic in Baghdad after the Mongol conquest due to the deterioration of the conditions in the city, overall they did *not* connect this disease outbreak in Baghdad in 1258 with the outburst of another epidemic in Syria and Egypt. Instead, we find several authors linking the slaughter of Baghdad’s inhabitants by the Mongols to the subsequent outbreak in Syria and Egypt using a miasmatic explanation, i.e. that the disease was caused by the corruption of the air engendered by the massacres in Baghdad.

Finally, while the subtle shift to more accurate, yet still succinct descriptions of plague symptoms (as well as the association of plague with the appearance of the bubo specifically in the armpits, along with inflammation) in Arabic medical commentaries largely in the second half of the thirteenth century may be important,^8^ we see *no* textual evidence for a connection between this shift and the 1258 epidemic in Syria and Egypt, and certainly no connection to the Mongol conquest of Baghdad. None of the thirteenth-century medical authors discussed by Fancy and Green explicitly state in any way that they had witnessed plague in Syria, Egypt, or Iraq. For example, in a recent article, Fancy introduced an important medical account of plague symptoms by the Damascene Ibn al-Nafīs (d. 1288, active, however, in Egypt); yet Ibn al-Nafīs’ description is based on an eyewitness account of a friend of his, and the latter witnessed and described a plague outbreak in Ethiopia (!), not in Syria or Egypt.^9^ Moreover, Fancy argues that these more accurate accounts appear already in the 1240s, namely more than a decade before the Mongols besieged Baghdad.^10^

## Plague during the Siege of Baghdad in 1258?

The linchpin for Fancy and Green’s analysis of plague outbreak after the Mongol siege over Baghdad and thus of the role that the Mongols had in the ‘spillover event’ that spread the bacterium *Yersinia pestis,* which causes plague, from its reservoir in the Tian Shan mountains^11^ is a passage from a short Persian chronicle attributed to the savant and official Quṭb al-Dīn Shīrāzī, titled *Akhbār-i Mughūlān* (‘Mongol News’), an edition of which was published only recently, in 2010.^12^ According to Green, this passage fits the pattern found in other contemporaneous textual accounts from China, according to which mysterious epidemics appeared shortly after Mongol sieges were lifted in China, as has been identified and discussed by Robert Hymes. He has further noted that Chinese medical writings record the appearance of a brand-new symptom—large purulent lumps or sores—in some descriptions of lethal epidemic outbreaks beginning in the Mongol incursions in the early to middle thirteenth century. This symptom fits plague buboes.^13^ Following Hymes, Green (and then Fancy and Green) suggest that *Akhbār-i Mughūlān* indicates the same ‘epidemic profile’. They hypothesize that the Mongol armies brought with them plague, likely transmitted via the rats infesting the grain supplies they carried from further east, which struck the local population after the siege was lifted. Here is the translation of the text by Shīrāzī that Green uses:

When Hulegu [*sic*] reached Baghdad the rest of his army, who were already in the city, was standing on the ramparts. Because a great assemblage of people, namely all the people of the Sawād, had come to the city before the Mongol army arrived, there was a great dearth, want, and scarcity of provisions in Baghdad. Pestilence struck and many people died. The number of deaths reached the point that the Ministry’s priority was to prepare the corpses and bury them. Meanwhile the situation deteriorated so much that the people of Baghdad could no longer cope with ablutions and burial of the dead, so the bodies were thrown into the Tigris.… Even when the army arrived, they were unable to cope with the situation.^14^

The original translation used by Green, however, is unclear as to *when* exactly the pestilence appeared in Baghdad. A close reading of the original Persian decisively shows that the author conveyed that the disease had struck the denizens of Baghdad *before* the Mongol forces arrived at the city’s walls. Here is our revised translation:

When Hülegü reached the gate of Baghdad, the rest of the army [the caliph’s army, according to prior passages] that had already been in the city [to defend the city from the oncoming Mongols] was standing on the ramparts*. Before the Mongol army arrived at Baghdad, epidemic* (wabāʾ*) had struck [the city] and many people died* [our emphasis]. This was due to the assemblage of many people [in the city], namely all the people of the Sawād,^15^ who had come to the city [seeking refuge from the Mongols], and there was therefore great dearth, want, and high prices. The number of deaths reached such a point that [payment] would be made immediately from the Treasury to prepare the corpses [for burial] and they were to be buried [without delay]. Meanwhile the situation deteriorated so much that the people of Baghdad could no longer cope with ablutions and burial of the dead, and they would throw bodies into the Tigris…. Even when [Hülegü’s] army arrived, they [the residents of the Baghdad] were still not clear of it [i.e. the pestilence and the burial of the dead].^16^

The Arabic/Persian term *wabāʾ* mentioned in *Akhbār-i Mughūlān* can refer to several contagious diseases, as it is a general term for deadly disease. It *may* refer to plague, although it usually appears more as an ‘umbrella term’ for epidemics. The term *ṭāʿūn* (below) specifically designates plague in Arabic and Persian sources.^17^ A correct understanding of the text, considering what comes before and after it, clearly shows that an epidemic broke out in Baghdad *before* the Mongol armies arrived and began to besiege the city. One can suppose that in the crowded and disturbed conditions in the city caused by an inflow of refugees from the surrounding countryside, without adequate food, living in squalor, and lacking basic sanitation for many days, and compounded by uncertainty and dread, the city was ripe for a mass outbreak of a disease of some type, or many types (dysentery, typhus, typhoid fever, etc.). This was often the case in sieges, or in this case, the days and weeks before a siege. Thus writes A.D. Lee, when discussing parallel events in late antiquity:

[M]alnutrition left individuals more susceptible to disease, and in the often-crowded conditions of a siege could spread rapidly, with fatal consequences for many. Problems with the appropriate disposal of bodies in turn exacerbated insanitary conditions, as many of those at Amida in 359 and Rome in 409 found to their cost.^18^

The same reservation must be made regarding the two other examples that Green provides for plague outbreak after Mongol sieges in the eastern Islamic world. She notes that a *wabāʾ* outbreak is reported in the Ismāʿīlī (‘Assassin’) fortress of Lanbasar (Lammasar) ‘at the end of the siege, in 1257’. She then proceeds to suggest that this outbreak ‘oddly’ parallels ‘the circumstances of the plague outbreaks in the sieges in China’.^19^ Yet, neither of the two sources referenced – Shīrāzī and Rashīd al-Dīn – indicate that the epidemic appeared after the siege was lifted; rather, both sources confirm that the fortress lasted under siege for about a year until an epidemic broke out, killing most of the besieged men.^20^ The outbreak is plainly mentioned by both authors as hastening the fall of the fortress to Mongol hands, and not as resulting from its fall. The same can be said about the earlier siege at Girdkūh in 1253. The outbreak of an epidemic during the Mongol campaign required the Ismāʿīlī ruler at Alamūt to send reinforcements to the castle to keep it from falling to the Mongol forces (which it managed to do for some time). Here, too, there is no evidence that the disease was brought by the besiegers.^21^

Another key piece of evidence adduced by Green (followed by Fancy and Green) is from the Syriac Aramaic chronicle by Bar Hebraeus (d. 1286). We offer a slightly different version from Budge’s translation used by Green:

And then, there was a very severe famine and a pestilence (*māwthānā*) in the land of Babylonia (Senʿār), Assyria (Āthūr), Mesopotamia (Bēth Nahrīn), Syria (Sūrīya), and Anatolia (Bēth Rūmaye), so a young pigeon for a sick man was sold in Damascus for 12 Nasiri dirhams.^22^

Fancy and Green see this as further proof of the spread of plague, which was earlier seen in Baghdad. We cannot agree, and state with a high degree of certainty that Bar Hebraeus is dealing here with a later event, some three or four years after the sack of Baghdad. The problem here is not the slight difference in translation, but the context. Looking at the entire paragraph, it is clear that the author is not at all referring to events right after the Mongol conquest of Baghdad, but has gone off on a tangent to discuss developments over several years in the city of Irbil (in northern Iraq of today) and the hill country to the north populated by Kurds (then, as today).

We need not render the entire paragraph, but merely note that before dealing with the ‘severe famine and pestilence’, it talks about ‘rebellious Kurds’ in a place that Budge gives as Jûlmarg. In Arabic this is the city of Jūlamark (modern Çölemerik in Turkey, about 150 km north of Irbil). Mamluk accounts, indeed, report a rebellion there, but in 1261–2 and not earlier.^23^ According to Bar Hebraeus, only then, at the earliest, did famine and pestilence break out in the regions listed by him. This fits very well with the parallel information from the Arabic sources that in late 1261, Syria suffered bad harvests (due, it is claimed by one author, to an outbreak of mice), and there was a shortage of food resulting in high prices.^24^ In short, Bar Hebraeus provides no support for a raging epidemic of any type in Iraq, upper Mesopotamia, and Syria in 1258.^25^ There may well have been a mass health emergency in these regions during this year (see below for Syria), but the Syriac version of Bar Hebraeus provides no evidence of this.

The third piece of evidence provided by the authors is taken from the anonymous Arabic chronicle of Baghdad in the seventh Hijri century (=1203–1300 CE), *al-Ḥawādith al-jāmiʿa*, which traditionally had been attributed to the Iraqi historian Ibn al-Fuwaṭī (d. 1323). In the description of the momentous events in 656 Hijri (=1258 CE), this is what we find relating to the massacre of the population and then the ensuing famine:

It was said that the number of dead in Baghdad was more than 800,000 souls, besides those children thrown into the mud, and those who died in the canals, wells and cellars, perishing from hunger and fear. Pestilence broke out among those who survived the massacre, from the stench of the dead, and from drinking water contaminated by corpses. People had much recourse to the smelling of onion due to [the reek] of the cadavers and the multitude of the flies, which filled the area, and alighted on foods, spoiling them.^26^

This seems straightforward to us: given all the dead bodies—victims of the massacre and the subsequent famine—and contaminated water and food, is it any surprise that an epidemic (or epidemics) broke out? Does one need to resort to the plague to explain the mass outbreak of disease here?

In summary, we see no clear evidence in these three passages that there was an outbreak of plague in Baghdad or adjacent regions to the north around the time of the Mongol conquest of the city in early 1258. The first passage, by Shīrāzī, mentions an outbreak of *wabāʾ* in the city before the beginning of the Mongol siege after many people from the countryside of Iraq fled to the city. The second passage, in the Syriac version of the chronicle by the Gregorius Bar Hebraeus, does not apply to 1258 at all, but to events of three or four years later. Finally, we have a description in the anonymous Arabic Baghdadi chronicle of mass disease in Baghdad among the local survivors after the Mongol conquest. Certainly, in both the first and third passages, the outbreak of epidemics is readily explained by crowded conditions, poor sanitation, a plethora of unburied corpses, and the resulting pollution of water (and maybe food) sources, all compounded by fear and uncertainty.

Whatever affliction spread among the city of Baghdad either before the Mongol armies arrived or immediately following the city’s surrender to Hülegü, we might expect that it would have also affected the Mongol armies. According to *Akhbār-i Mughūlān*, after Hülegü’s army returned from Baghdad to Azerbaijan in the year 656 Hijri (1258), ‘the weather had become warm, and a great stench (*ʿufūnat*) [i.e. corrupted air] entered the people’s brains. [Subsequently] an epidemic (*wabāʾ*) struck, and most of the Mongol army was afflicted, and many died’. The source further reports that Hülegü himself became afflicted, but after twenty days recovered.^27^ Did this epidemic have anything to do with the disease that afflicted the residents of Baghdad before (and likely during) Hülegü’s siege of the city? Possibly, although the affliction might have been related to the heat wave reported in the account. In either case, there is no evidence that we are dealing with plague. Rather, it is important to note that *Akhbār-i Mughūlān* reports a disease that afflicted the Mongol forces after Baghdad’s fall, making unlikely the possibility that the Mongols served as vectors that delivered the pandemic to Baghdad in the first place.

Another problem with Fancy and Green’s theory relates to the spread of the disease after the fall of Baghdad. Right after the above-cited description in *al-Ḥawādith al-jāmiʿa*, the anonymous Arabic chronicler of Baghdad continues:

The people of Ḥilla, Kūfa and Sīb [all in southern Iraq] brought to Baghdad food, from which the [local] population took sustenance. These [people from the nearby cities] took for the prices of [this food] precious books, inlayed copperware, and valuable furniture. A large number of these people were enriched in this way.^28^

It seems, then, that visitors from other cities in Iraq, who immediately exploited the desperate situation of the surviving Baghdadis, remained untouched by the ravages of plague that supposedly affected the last mentioned. How so?

## A Re-examination of the Mamluk Sources for an Epidemic in Syria and Egypt in 1258

Beyond these three accounts, the Arabic sources written at the close of Ayyubid rule in Syria (1260 CE) and in the Mamluk Sultanate (in Egypt from 1250–1517; in Syria 1260–1516) offer some evidence for epidemics in Baghdad, as well as for Syria and Egypt in 1258, after the fall of Baghdad. In a table, and then in their discussion, Fancy and Green note sixteen sources conveying this material,^29^ concluding that ‘the role of epidemic disease in the Mongol attacks was commonly known among chroniclers’.^30^ They further argue that a peculiar historiographical ‘erasure’ took place, in which later, post-Black Death authors failed to identify the pandemic’s thirteenth-century episode.^31^

To prove this ‘erasure’ of evidence, they put together an impressive survey of a large corpus of passages, usually in chronicles that are particularly verbose for the events of the *annus horribilis* of 1258.^32^ Their argument here is based on a division of these sources by generations.^33^ This division makes little sense to us, and we argue instead that a better approach is identifying four *Urtexte* from historians, apparently all contemporary to the events of that year, and then, observing how later authors copied, summarized, or subtly changed these earlier observations. We have thus concentrated on these original accounts, keeping our eyes open for novel information that might have been added by subsequent authors. To keep an open mind, we have not translated the two Arabic words *wabāʾ* and *ṭāʿūn.* ^34^

When looking at these sixteen texts (and several more that we have added), we can identify four separate accounts that are passed down from one historian to another over a period of almost two centuries, along with a couple of ‘dead ends’ that were not reproduced by later writers. Here the authors demonstrate, as noted above, a well-known phenomenon in the historiography of the Ayyubid and Mamluk eras: later writers cite passages (at times, also the names of the earlier authors are noted), sometimes almost word for word, but often summarized (occasionally in a rather terse way), and once in a while, new information is added, usually tacitly.^35^ Actually, as we will see below, all four original accounts are repeated with few additions, and if anything, they generally get shorter over time. On the other hand, we do have one example where a later writer renders a much fuller text by an unnamed source, but we will suggest that this particular passage probably harks back to the time of the events themselves and is only partially rendered by historians of the next generation.

Briefly, the four original accounts are as follows:

1. *Wabāʾ* broke out in Baghdad following the fall of the city, and high numbers for the dead are provided. The original account here is the passage cited above from the anonymous (pseudo-Ibn al-Fuwaṭī) Baghdadi chronicle *al-Ḥawādith al-jāmiʿa.*
2. An epidemic (referred to as both *ṭāʿūn* and *wabāʾ*) erupted in Syria and Egypt in 1258. The original author of this account is Ibn Wāṣil. He himself came down with it in Cairo but recovered. There is a quick survey of mention of *ṭāʿūn* from a medical text and early Islamic history. The Ayyubid prince al-Nāṣir Dāwud got sick and died; according to the source, this was *ṭāʿūn.*
3. In the aftermath of widespread disease (*maraḍ*) and *wabāʾ* in Syria, large numbers died in Aleppo and Damascus, but in the former we get an exact daily figure. We learn of the suffering of the inhabitants, along with the high prices and shortages in both cities.
4. Mention *en passant* of ‘sickness’ in Egypt, as part of the biography of a poet and courtier there.

**Account 1:** We start with *al-Ḥawādith al-jāmiʿa.* ^36^ This passage has been cited above, but now we are examining its historiographical impact; we provide here a fuller rendition than above:

The dead bodies lay in mounds in the alleys and market. Rain fell upon them, and the horses trampled on them. Their shapes were disfigured, and they became an example (i.e., embedded in their memory) to whomever saw them. Then an *amān* (amnesty) was announced. Those who survived came out [from hiding], but their faces went white and they were shocked by the horror that they saw, that could not be described in words. They were like the dead that had emerged from the graves on the day of resurrection, [suffering] fear, hunger and cold…^37^

It was said that the number of dead in Baghdad was more than 800,000 souls, besides those children thrown into the mud, and those who died in the canals, wells, and cellars, perishing from hunger and fear. *Wabāʾ* broke out among those who survived the massacre, from the stench of the dead, and from drinking water contaminated by corpses. People had much recourse to the smelling of onion due to [the reek] of the cadavers and the multitude of the flies, which filled the area, and alighted on foods, spoiling them. The people of Ḥilla, Kūfa and Sīb [all in southern Iraq] brought to Baghdad foods, from which the [local] population took sustenance. These [people from the nearby cities] took for the prices of [this food] precious books, inlayed copperware, and valuable furniture. A large number of these people were enriched in this way.^38^

Later authors summarized this passage. Ibn Shākir al-Kutubī (d. 1363), author of *ʿUyūn al-tawārīkh*, gives a short rendition of both these passages:

It is said that the number of dead in Baghdad was more than 1,800,000 souls [N.B. 800,000 has become 1,800,000], besides those children thrown in the mud, and those who died in the canals, the wells and cellars, perishing of hunger and thirst. *Wabāʾ* broke out among those who survived the massacre, from the stench of the dead, drinking water contaminated by corpses, and the multitudes of flies, which filled the area, and alighted on foods, spoiling them.^39^

A parallel, but somewhat different, text is from Ibn Kathīr’s (d. 1373) *al-Bidāya wa’l-nihāya*:

People disagreed about the amount of those who died in Baghdad among the Muslims in this event. It was said 300,000, [also] it was said 1,800,000, and [finally] it was said that the dead reached 2,000,000 … An amnesty (*amān*) was announced in Baghdad, and those who had been under the ground emerged from underground storerooms, subterranean water cannels and tombs, as if they were the dead when exhumed from their graves. One would disown the other: the father did not know his son, and one did not recognize his brother. A strong *wabāʾ* afflicted them, and they died, joining those who had preceded them in death.^40^

This version is rendered in a slightly shorter form by al-ʿAynī (d. 1451) in his *ʿIqd al-jumān.* ^41^ Al-ʿAynī does not name his source, as he often does, but elsewhere in his account of the conquest of Baghdad, he cites Ibn Kathīr, so this later writer is most probably al-ʿAynī’s direct source here as well. Other authors give even shorter versions, concentrating on the numbers who died in Baghdad.^42^ Al-Dhahabī (d. 1348) provides in *Taʾrīkh al-islām* the figure of 300,000 dead, not an unrealistic number.^43^ We will return below to other passages by this last-mentioned author.

In summing up this particular *Urtext* and its derivative passages, we can first note again that there is nothing here indicating plague, but rather diseases caused by contaminated water and other conditions. We can also note for now that the graphic details presented by the anonymous author gradually disappear in most of the works of later historians (al-ʿAynī is an exception). What remains is the focus on the numbers of the dead, which are rendered in an exaggerated manner; al-Dhahabī (followed by Ibn Taghrī Birdī), however, returns us to reality by citing ‘just’ 300,000 dead. Finally, neither the anonymous writer nor any of the later authors who used his account, either directly or indirectly, linked this disease outbreak in Baghdad to the epidemic that struck Syria and Egypt later in 1258. Other authors, however, did imply a relationship between the epidemic that spread in Syria and Egypt and the Mongol assault on Baghdad. The first author to do so appears to have been Ibn Wāṣil (d. 1298),^44^ to whom we now turn.

**Account 2:** In his chronicle *Mufarrij al-kurūb,* ^45^ Ibn Wāṣil (d. 1298) reports first how *tāʿūn* erupted in Syria and Egypt, in the aftermath of the Mongol conquest of Baghdad. This author himself came down with this disease in Cairo, but recovered. The author relates how the Ayyubid prince al-Nāṣir Dāwūd got sick and died near Damascus.^46^

*Ṭāʿūn* spread throughout all of Syria and Egypt, and to other places. Corruption (*fasād*)^47^ caused this, leading to the affliction of the *wabāʾ* and the changing of temperaments (*amzija*). I saw the most amazing thing in Egypt: in Bilbis high fever and coughing hit, so that nearly no one was spared it, but in Cairo no one was affected. Then, two days later, something like this happened in Cairo. At that time, I was staying in Giza; I rode to Cairo, and I found the matter (i.e., the sickness) there afflicting almost all the people of Cairo. I returned to Giza, and just settled in when I came down with it too, and it spread to the people of Giza. In most cases the coughing lasted more-or-less three days. Most people survived.^48^ It passed from one town to the next, until it gradually reached the farther regions of Egypt.

Galen described it in a like matter: there was slaughter (*malḥama*) in Greece, and *wabāʾ* afflicted the land of Nubia after a while. ʿAbd Allāh b. al-Faḍl, one of al-Malik al-Nāṣir Dāwūd‘s entourage, reported:

When the *wabāʾ* and *ṭāʿūn* intensified in the aftermath of the Mongol conquest of Baghdad, we were dismayed (*tasakhkhaṭnā*) by it. Al-Malik al-Nāṣir said to us: ‘Don’t be dismayed.’ As for the *ṭāʿūn*, when it struck ʿAmwās during the caliphate of ʿUmar b. Khaṭṭāb (r. 634–44)—may God be satisfied by him—he said to some of the people: ‘This is the punishment of God (*rijz*); this is the disaster (*ṭūfān*) that was set upon the Children of Israel.’^49^

This reached Muʿādh b. Jabal (d. 639),^50^ may God be satisfied by him, who functioned as preacher (*khāṭib*) among the [Muslim] people, who said:

O people! Why do you call to your Prophet—may God’s prayer and peace be upon him—and for the mercy of God from the suffering [of the pestilence]. Think that the *ṭāʿūn* is the disaster (*ṭūfān*) that was set upon the Children of Israel.^51^ The *ṭāʿūn* is the mercy of God, who forgives you, and the prayer of your Prophet upon you. O God, give to the family of Muʿadh their complete portion.

[ʿAbd Allāh b. al-Faḍl] said: ‘Al-Malik al-Nāṣir told us about the death of Muʿādh, his son and his family by *ṭāʿūn.*’ Then al-Malik al-Nāṣir prayed, saying: ‘O God, make us like them and make our lot what you gave them.’ He woke up the next morning or afterwards, afflicted by the *ṭāʿūn* (*wa-aṣbaḥa … maṭʿūn^an^*).^52^ I was absent. When I heard of his illness, I came to him. He complained [of a pain] like a stab (*ṭaʿn*)^53^ from a sword in his left side that prevented him from lying down. At dawn of Thursday, this eased up, and he lay down and awoke with a fever.

His son al-Malik al-Muẓaffar Shihāb al-Dīn Ghāzī, who now lives in Cairo, related that [al-Nāṣir Dāwūd] slept between the [first] two prayers, and then he woke up and said: ‘I saw my left side say to my right side, “My turn has come and I put up with it, and tonight your turn will come, and you will put up with it, as I did.”’ When evening came, he complained of a slight pain on his right side, and this began to get worse. We knew that this was *ṭāʿūn.* I was with him between the two prayers, and his strength was diminishing, when sleep overtook him. He woke up, and he was shaking. He motioned for me, and I drew near to him. He said, ‘I saw the Prophet—may God’s prayer and peace be upon him—and al-Khiḍr^54^—peace upon him—came to me and sat with me; then they left.’ At the end of the day he said, ‘I have no hope, so prepare my funeral.’ I wept and all those present wept … He entrusted his family and children to me. Then at night, he became weaker… [Details of a further dream, and his funeral; death not explicitly mentioned].

As Fancy and Green note, Ibn Wāṣil provides in his account a miasmatic explanation based on Galen for the disease outbreak in Syria and Egypt.^55^ According to this theory, corrupted air due to changes in the seasons or the temperatures, or from noxious vapours and unpleasant stenches from fires, stagnant waters, or the rotting of unburied corpses, can cause contagious diseases. The polluted air is inhaled by individuals corrupting their internal organs and spirit. Moreover, this corrupted air can spread quickly over a large distance with the winds. ^56^ As the reader can see, however, while Ibn Wāṣil mentions the miasmatic explanation for the epidemic in Syria and Egypt at the beginning of the account (due to corruption, *fasād*), he does *not* make the connection with the Mongol attack on Baghdad in this long passage explicit, just noting further along: ‘When the *wabāʾ* and *ṭāʿūn* intensified in the aftermath of the Mongol conquest of Baghdad, we were dismayed by it.’^57^

A number of later writers provide fairly short versions of this long and detailed account, centering around the death of al-Malik al-Nāṣir Dāwūd.^58^ Worth mentioning first is al-Dhababī (d. 1348), who cites by name Ibn Wāṣil:^59^

The *ṭāʿūn* spread in Syria, after it had finished in Iraq. Al-Nāṣir [Dāwūd] was ‘stabbed’ (*ṭuʿina*, implying he was affected by *ṭāʿūn*). Ibn Wāṣil said that it spread in spite of the distance from Baghdad. Galen related that after the slaughter (*malḥama*) in the land of Greece, there was *wabāʾ* because of it in the land of Nubia, in spite of the distance.

Then he gives a shorter version of al-Nāṣir Dāwūd getting sick, telling his comrades not to complain about the *wabāʾ* (N.B., compare with his source, Ibn Wāṣil, who writes *wabāʾ* and *ṭāʿūn* here), the beginning and intensification of his sickness, and death. A brief version of this story is also related in the *Mukhtaṣar* by the Ayyubid prince and scholar Abū al-Fidāʾ (d. 1331),^60^ who generally summarizes Ibn Wāṣil’s chronicle for the decades before he himself was a contemporary of events. He, in turn, is apparently followed by Ibn al-Wardī (d. 1348-9).^61^ Abū Shāma (d. 1268), a contemporary resident of Damascus, notes briefly the death of al-Nāṣir Dāwūd, but does not mention his disease.^62^

What do we learn from the above passage from Ibn Wāṣil and its derivatives? The original author reports that *ṭāʿūn* spread in Syria and Egypt, and he himself got it in Cairo. He recovered, however, as did most people. A prominent and well-respected Ayyubid prince in Syria, al-Nāṣir Dāwūd, who, however, was no longer enjoying any political power, came down with *ṭāʿūn*, eventually dying. We can note that Ibn Wāṣil and his ‘epigoni’ go back and forth between the terms *ṭāʿūn* and *wabāʾ*, when describing this disease, suggesting that perhaps there was no clinical distinction between them in their minds.

Before moving on, we can also observe that to our understanding there is no symptom in Ibn Wāṣil’s report that leads us to consider that we are dealing here with plague. The disease that broke out in Egypt, which he himself came down with, was characterized by fever and coughing, with a low deathrate. Fever is certainly not unique to plague, nor is coughing. These suggest other diseases, influenza perhaps (see in next section for more on this). Beyond the use of the word *ṭāʿūn* (and the passive participle and verb derived from its root), there is little to tie this disease to plague. We, therefore, should consider the possibility that al-Nāṣir Dāwūd’s affliction was identified with *ṭāʿūn*, not based on symptoms, but rather to assign al-Nāṣir Dāwūd the same idea of divine merit (‘God’s mercy’) and martyrdom that Islamic traditions associated with those afflicted with plague in the seventh-century epidemic at ʿAmwās.^63^

While Fancy and Green indeed observed that Ibn Wāṣil was the first chronicler to employ *ṭāʿūn* with regard to the disease in Syria and Egypt (as well as the first to use the miasma explanation),^64^ they did not stress the fact that he was also *the only source* for all subsequent uses of the term *ṭāʿūn* for identifying the epidemic in Syria and Egypt.

**Account 3:** Ibn Wāṣil (d. 1298), al-Yūnīnī (d. 1326), and al-Dhahabī (d. 1348) provide additional important information regarding *wabāʾ* in Syria, in Damascus and Aleppo in 1258, in accounts that share many elements, but are not identical. They describe the deathrate in Aleppo, the high prices and shortages in both cities, and overall suffering. In addition, al-Dhahabī also unequivocally notes the miasmatic link between the Mongol slaughter of Baghdad’s denizens and the epidemic in Syria. These accounts have been surveyed and discussed by Fancy and Green, and they suggest a reliance of al-Yūnīnī and al-Dhahabī on Ibn Wāṣil.^65^ On the contrary, we propose that these three historians share a hitherto unknown common source. In fact, we have uncovered the fullest version of this passage, which we now present, and suggest that it represents a contemporary account, as well as the common source for all three previously mentioned reports. Our source is an unpublished manuscript volume, found in the Vatican Library, from *Taʾrīkh al-duwal wa’l-mulūk* by the Egyptian historian Ibn al-Furāt (d. 1405). The translation is found below, while the Arabic text is in the Appendix:

One of the historians said that the sickness (*maraḍ*) and *wabāʾ* afflicted the people in Syria after the taking of Baghdad, and so that the people attained [access to] pharmacists only with great effort and difficulty. The pharmacists were enriched, medicines were used up, and physicians and bloodletters (or cuppers) were hardly found. Making a living was impossible, and there was no request for merchandise. The people acted towards the pharmacists as they would towards bakers during a famine. The situation lasted days. As for Aleppo, death there was greater: news arrived that every day 1200 funerals left the city. In Damascus, many people died on the street and in the hospitals, and both Qur’an readers and corpse washers were hardly found. Most of the people of the city were afflicted by fever and coughing [as if it was] one disease (*maraḍ wāḥid*). A number of notables died. As for those who did not have [medical] care, a large number [died among them]. At this time, a slice of green melon^66^ was sold in Damascus for a dirham and a *raṭl* of *tamar hindī* ^67^ went for 60 dirhams. As for pullets (*al-farārīj*, i.e. young chickens), they were not to be found, but if one the size of a bird was found, they were selling (it) for one dirham, and this became three or four dirhams [for a bird]. As for Aleppo, the price of every pullet there reached 10 dirhams. A short [description] absolves [us] of a detailed one. God knows best.^68^

Unfortunately, Ibn al-Furāt, who is usually scrupulous in citing his sources by name, leaves us in the dark here; yet this source was likely a contemporary or near contemporary of the events. It is clear, however, that he has brought us the fullest text for the of this particular chain of accounts, and thus we propose that it represents the common source for the versions by Ibn Wāṣil, al-Yūnīnī, and al-Dhahabī.^69^ There is no a priori reason to reject the contemporary nature of Ibn al-Furāt’s account, and the fullness of its description adds to the consideration of being the earliest version of this particular account.

Ibn Wāṣil, whom we have seen was a contemporary of these events, has the following to say:

In this year, that is to say 656 [AH, i.e. 1258], the *wabāʾ* got worse in Syria, especially in Damascus, so that scarcely a washer of corpses could be found. The numbers who perished among the city’s population could not be counted. The price of pullets became dear, and they were not to be found. If one was found, it was quite small, and was sold for two or more dirhams.^70^

Being a contemporary does not mean that everything Ibn Wāṣil wrote was in ‘real time’: the long chronicle *Mufarrij al-kurūb* was begun in 1272 and completed only in 1285.^71^ This would have been plenty of time for another account of this outbreak of *wabāʾ* to have appeared and be summarized by Ibn Wāṣil. The fact that he placed this short passage far away from his long personal account (see Account 2 above), also leads one to think that it was not necessarily originally his own eyewitness version.

Al-Yūnīnī, who would have been 16 years old when the *wabāʾ* broke out, provides another version in his *Dhayl mirʾāt al-zamān.* However, rather than relating personal memories, al-Yūnīnī too probably summarized the long account that Ibn al-Furāt conveyed:

In this year, *wabāʾ* intensified in Syria, and an uncountable number of people of Damascus died. [The price] of pullets and other [foodstuffs] that serving the sick shot up. A *raṭl* of *tamar hindī* cost 60 dirhams and the slice of a green melon was a dirham.^72^

From here we can go directly to his younger contemporary, al-Dhahabī, in his *Taʾrīkh al-islām*:

And during this year [AH 656] the *wabāʾ* got worse in Syria, and [so many] people died, so that it is said that in Aleppo every day 1200^73^ funerals set out. As for Damascus, there was unlimited and indescribable sickness (*al-maraḍ*) and the pharmacists were enriched, medicines were used up, and physicians were hard to find. Pullets were sold in Damascus for three dirhams, and in Aleppo for 10 dirhams. The beginning of the *wabāʾ* was in Jumādā I (May-June 1258), due to the corruption (*fasād*) of the air in the slaughter (*malḥama*) at Baghdad. ^**74**^

These two texts, like that of Ibn Wāṣil, share data and formulations, but each is different. Again, at the risk of overstating the case, the reliance on a common source—evidently preserved by Ibn al-Furāt—seems very likely. There is here, however, an interesting and unique piece of information conveyed by al-Dhababī: ‘The beginning of the *wabāʾ* was in Jumādā I (May-June 1258), due to the corruption (*fasād*) of the air in the slaughter (*malḥama*) at Baghdad.’ This is certainly not in Ibn al-Furāt’s longer text, where it is only written that ‘the sickness (*maraḍ*) and *wabāʾ* afflicted the people in Syria after the taking of Baghdad.’ It is clear, however, that the terms ‘corruption’ (*fasād*) and ‘slaughter’ (*malḥama*) hark back to Ibn Wāṣil’s long account discussed in the previous section. Al-Dhahabī has deliberately, and seamlessly, combined elements from two accounts—the ‘Urtext 3’ preserved evidently by Ibn al-Furāt and ‘Urtext 2’ from Ibn Wāṣil’s chronicle. Writing at least half a century after the events he is describing, al-Dhahabī might have believed in the miasmatic connection between the slaughters in Baghdad and later pandemics in Egypt and Syria (which he refers to as both *ṭāʿūn* and *wabāʾ*). This, however, is far from compelling evidence that we are dealing with the same disease, let alone that it was transferred from Iraq to Syria, and then to Egypt.^75^

One matter raised by Ibn al-Furāt, however,^76^ needs to be addressed: the occurrence of ‘fever and coughing’ (*bi’l-ḥummay wa’l-suʿāl*) in Damascus might not be just some form of influenza or another ‘regular’ respiratory disease, but rather symptoms of pneumonic plague. About the latter, we can cite from Gage and Beard (insertions in square brackets are ours):

Pneumonic plague is the most rapidly developing and life-threatening form of plague. The incubation period for primary pneumonic plague is usually 2–5 days (range 1–6 days). Illness onset is most often sudden, with chills, fever, body pains, headache, weakness, dizziness and chest discomfort. Cough, sputum production, increasing chest pain, tachypnea [rapid breathing] and dyspnea [shortness of breath] typically predominate on the second day of illness; hemoptysis [spitting up blood], increasing respiratory distress, cardiopulmonary insufficiency and circulatory collapse can also occur. The sputum of primary plague pneumonia patients is typically watery or mucoid, frothy and blood tinged, and can be bloody.^77^

We do not have detailed symptoms from any of the sources that can confirm or rule out the above in either Aleppo or Damascus. On the other hand, there was a high deathrate for at least a few days in both cities (with explicit numbers in Aleppo). One can only suggest that had there been a mass outbreak of pneumonic plague in these cities, the deathrate would have been even higher, and the symptom descriptions so much more vivid. The use by all sources of the term *wabāʾ*, even by the ‘*ṭāʿūn* informed’ al-Dhahabī, also inclines us not to see plague here, pneumonic or otherwise. We are still on safe ground when we claim that another type of respiratory diseases was wreaking havoc here.^78^

Let us sum up this section as follows: ‘sickness and *wabāʾ*’ are mentioned in this Urtext and its derivative passages, not *ṭāʿūn* (except for al-Dhahabī, clearly influenced by Ibn Wāṣil). The symptoms were fever and coughing (and no more), again reminiscent of influenza, not plague. The number of dead was indeed high. Medicine, medical and funeral experts, and other supplies were scarce and thus expensive. This was a difficult situation, but it was not directly connected to the Mongol conquest of Baghdad earlier in the year, nor caused by plague.

**Account 4:** Another transmission thread about the epidemic in Egypt is provided in the biographical collection *Wafāyāt al-aʿyān* by Ibn Khallikān (d. 1282). In the biography of one Bahāʾ al-Dīn Zuhayr b. Muḥammad al-ʿAtaqī,^79^ a poet and courtier who died during an epidemic in the year 1258 in Egypt, we learn that:

A great sickness (*maraḍ*) fell on Cairo and Fustat (*al-Qāhira wa’l-miṣr*), from which few were spared. It began on Thursday, 24 Shawwāl, the year 656 (AH, October 30, 1258). The above-mentioned Bahāʾ al-Dīn was one who suffered from it. He continued in this way for a few days, then dying before the evening prayer on Sunday, Dhū al-Qaʿda of this year … I did not pray for him, as I was myself preoccupied with the disease.^80^

Some fourteenth century writers summarize this information: al-Dhahabī and al-Ṣafadī briefly note that after Bahāʾ al-Dīn returned to Cairo following a stay in Syria, ‘he was sick for a few days with *wabāʾ* and died.’^81^ Clearly, *maraḍ* and *wabāʾ* were equated by contemporary and slightly later contemporaries.

To all the above we might add some independent pieces of testimony, none of which seem to have been picked up by other writers. Another contemporary was the legal scholar and historian Abū Shāma (d. 1268), a resident of Damascus, who wrote tersely:

During the time of Spring [656 AH/1258 CE], there was a lot of *wabāʾ*, and it was stranger than what was [usually] written in history. The disease (*maraḍ*) spread among the people, and there was much death.^82^

Finally, the historical work by the famous Ibn Khaldūn (d.1406), *Kitāb al-ʿibar*, is unfortunately not very helpful here, writing only that ‘the Mongols had already conquered Baghdad, and then withdrew. In some of the villages of Damascus, [people] died.’^83^

## Conclusions

Rereading the sources surveyed by Green and Fancy, we have found no evidence in the various texts that would tie the various epidemics/pandemics in Iraq, Syria, and Egypt to plague, supposedly brought unintentionally by the advancing Mongol army that besieged Baghdad in early 1258. We have seen that the Persian-language text attributed to Quṭb al-Dīn Shīrāzī clearly stated that *wabāʾ* broke out in Baghdad before the Mongols conquered it. Another account, the anonymous (pseudo-Ibn al-Fuwaṭī) Arabic text from Baghdad describes the outbreak of *wabāʾ* after the taking of the city by the Mongols, but in circumstances that clearly imply that its origins were the terrible conditions of the survivors.

Looking at the many late Ayyubid and Mamluk historians, we have identified four original and independent accounts that we have referred to as *Urtexte.* By identifying these original versions and then following how later authors incorporated and modified them, we have observed some important points: 1) A group of historians reported the outbreak of an epidemic in Baghdad after the Mongol conquest due to the deterioration of the conditions in the city. They seem to be based on, or derived from Urtext 1, the anonymous [pseudo-Ibn al-Fuwaṭī] chronicle *al-Ḥawādith al-jāmiʿa*, but they **did not** connect this disease with the outbreak of another epidemic in Syria and Egypt in the following years. 2) The only accounts to use the term *ṭāʿūn* are all based on Urtext 2, the long passage by Ibn Wāṣil. We suggested above that Ibn Wāṣil may have identified al-Nāṣir Dāwūd’s affliction with *ṭāʿūn* (plague) not based on his symptoms, but in attempt to credit al-Nāṣir Dāwūd with the death of a martyr, reminiscent of those who had died of plague in seventh-century ʿAmwās. 3) Two accounts probably based on Urtext 3 (contained in Ibn al-Furāt’s chronicle) suggest that it was the slaughter of Baghdad’s inhabitants that led to the subsequent outbreak in Syria and Egypt. One is the passage by Ibn Wāṣil, explaining that the corruption of airs had caused the epidemic in Egypt and Syria, and then further along alludes, or rather ‘hypothesizes’, that Mongol conquests related to this by referencing a tradition by the Greek physician Galen. This insinuation of a miasmatic connection was more clearly and decisively articulated by a later author, al-Dhahabī. However, let us remember that the unidentified historian cited by Ibn al-Furāt only writes that ‘One of the historians said that the sickness and *wabāʾ* afflicted the people in Syria after the taking of Baghdad’; no miasmatic connection here. Thus, the majority of Mamluk authors did not connect reports on a disease outbreak in Baghdad with the epidemic in Syria and Egypt. In short, there is simply no reason to conclude that ‘the role of epidemic disease in the Mongol attacks was commonly known among chroniclers’ as Fancy and Green argue.^84^

We have found the use—interchangeable at times—of three terms used to describe the various epidemics in Syria and Egypt in 1258: *wabāʾ*, *maraḍ,* and *ṭāʿūn.* While the last mentioned was also applied to plague, there is nothing concrete in the various passages that we have examined here (mostly also presented in Fancy and Green’s paper) to indicate the epidemics in in Syria and Egypt were plague. Secondly, when symptoms are described in these passages, we encounter fever and coughing – indicating perhaps some type of respiratory-centered ailment, such as influenza; there is no indication in the Arabic sources of the presence of extreme symptoms of either bubonic or pneumonic plague.

In short, when looking closely at the numerous passages, as we have done, and then integrating these with a critical reading of the sources describing epidemic disease in Baghdad during and after the conquest (in section 1 of this article), we have come to the conclusion that there is no cogent historical argument for the presence of plague in Iraq, and then in Egypt and Syria in the year 1258, the year that Baghdad was conquered by the Mongols. The connection between the epidemic in Baghdad and that in Syria and Egypt is questionable, and certainly not as obvious as presented by Fancy and Green.^85^

Fancy and Green have attributed the appearance of detailed and relatively accurate descriptions of plague symptoms in Arabic Ḥadīth and medical commentaries from the second half of the thirteenth century to this supposed thirteenth-century plague event in Iraq, then Syria and Egypt.^86^ This is, of course, an important and interesting matter, but at this point it appears to be far-fetched to connect this shift specifically to a supposed 1258 epidemic in Syria and Egypt, and certainly not to the Mongol conquest of Baghdad. In fact, that the plague symptoms were well known in post-1258 Egypt and Syria, yet none of the medical authors connect them to the 1258 events, only strengthens our argument that there is no relationship between the two phenomena.

In conclusion, our close reading of the texts brought by Fancy and Green (along with other passages) convincingly showed that there is no evidence that connects the epidemics in Baghdad, Syria and Egypt in 1258 to the Black Death. Nor does the recent paleogenetic evidence support a thirteenth-century outbreak of the plague in Central Asia or elsewhere. The Mongol conquest of Baghdad and the Black Death are both medieval catastrophes that accumulated mythical dimensions, and linking the two is very tempting. The textual evidence, however, does not support this connection.

## Footnotes

1

Monica H. Green , ‘The four black deaths’, American Historical Review, 125, 5 (2020), 1600–831; Nahyan Fancy and Monica H. Green , ‘Plague and the fall of Baghdad (1258)’, Medical History, 65, 2 (2021), 157–77. Green repeats many of these arguments also in: Monica H. Green , ‘Putting Asia on the black death map’, The Medieval Globe 8, 1 (2022), 61–89 .

2 Fancy and Green, *op. cit.* (note 1), 167–9; Nahyan Fancy , ‘Knowing the signs of the disease: plague in the Arabic medical commentaries between the first and the second pandemics’, in Lori Jones and Nükhet Varlık (eds.), Death and Disease in the Medieval and Early Modern World: Perspectives from Across the Mediterranean and Beyond (New York: Medieval Press, 2022), 35–66 .

3

Maria A. Spyrou , Lyazzat Musralina , Guido A. Gnecchi Ruscone , et al. ‘The source of the Black Death in fourteenth-century Central Eurasia’, Nature, 606 (2022), 718–2435705810 10.1038/s41586-022-04800-3PMC9217749. https://doi.org/10.1038/s41586-022-04800-3; see also Philip Slavin , ‘Death by the lake: mortality crisis in early fourteenth-century Central Asia’, Journal of Interdisciplinary History, 50 (2019), 59–90 ; Philip Slavin , ‘A rise and fall of a Chaghadaid community: demographic growth and crisis in ‘late-medieval’ Semirech’ye (Zhetysu), *circa* 1248–1345’, *Journal of the Royal Asiatic Society*, Ser. 3, 32 (2022), 1–32 .

4 Spyrou *et al.* , *op. cit.* (note 3), 718–24.

5 Fancy and Green, *op. cit.* (note 1), 157.

6

Michael W. Dols , The Black Death in the Middle East (Princeton: Princeton University Press, 1977), 68–74 , 315–9. The World Health Organization gives the following symptoms for Bubonic Plague: ‘People infected with plague usually develop influenza-like symptoms after an incubation period of 3–7 days. Symptoms include fever, chills, aches, weakness, vomiting and nausea. There are 3 main forms of plague. Bubonic plague is the most common and is caused by the bite of an infected flea. The plague bacillus, *Y. pestis*, enters at the bite and travels to the nearest lymph node to replicate. The lymph node becomes inflamed, tense and painful, and is called a bubo. With advanced infections, the inflamed lymph nodes can turn into suppurating [i.e., pus filled, festering; the authors] open sores. Bubonic plague cannot be transmitted from human to human.’ https://www.who.int/health-topics/plague#tab=tab_2, accessed on 26 February 2023.

7 Fancy and Green, *op. cit.* (note 1), 159.

8
*Ibid.*, 168–9.

9 See further: Fancy, ‘Knowing the signs of the disease’, *op. cit.* (note 2), 56–9. Fancy demonstrates that almost all post-1240 descriptions of plague symptoms were textually interlinked. Thus, Shīrāzī drew both on the works of Ibn al-Nafīs and Ibn al-Quff; *ibid.*, 62–4. It is certainly possible that one of these post-1240 authors witnessed plague, but since they traveled around, one cannot conclude with any certainty when and how they encountered it.

10 Fancy, ‘Knowing the signs of the disease’, *op. cit.* (note 2), 36, 55ff.

11 Fancy and Green, *op. cit.* (note 1), 158.

12 Actually, this translation of the title was applied by the original translator, George Lane, in a paper first describing the work to a wider scholarly audience: ‘Mongol News: The Akhbār-i Moghulān dar Anbāneh Quṭb by Quṭb al-Dīn Maḥmūd ibn Masʿūd Shīrāzī’, *Journal of the Royal Asiatic Society*, 3rd ser. 22, 3–4 (2012), 541–59. Perhaps less catchy, but more accurate would be ‘[Historical] information of the Mongols’ or just plain ‘The history of the Mongols’.

13

Robert Hymes , ‘A hypothesis on the East Asian beginnings of the Yersinia pestis polytomy’, The Medieval Globe, 1, 1–2 (2014), 285–308 ; Robert Hymes , ‘A tale of two sieges: Liu Qi, Li Gao, and epidemics in the Jin-Yuan transition’, Journal of Song-Yuan Studies, 50 (2021), 295–363 . In his most recent article, Hymes also notes, however, the discrepancies between what he identifies as the earliest potential references of a ‘Mongol-associated epidemic in or near China’ and Green’s hypothesis concerning the origin of plague in the Mongol conquest of the Qara Khitai and in the Tian Shan region. He suggests that ‘further searching might even still locate surviving pre-polytomy strains in those more eastern areas’. Robert Hymes , ‘Buboes in thirteenth-century China: evidence from Chinese medical writings’, The Medieval Globe, 8, 1 (2022), 3–59 (quote on page 51).

14 Quṭb al-Dīn Shīrāzī, *The Mongols in Iran: Quṭb al-Dīn al-Shīrāzī’s Akhbār-i Moghūlān*, trans. and ed. George Lane (Abingdon: Routledge, 2018), 52.

15 The rich countryside around Baghdad.

16 Quṭb al-Dīn Shīrāzī*, Akhbār-i Mughūlān dar Anbānah-yi Mullā Quṭb*, ed. Irāj Afshār (Qum: Kitābkhānah-i Buzurg-i Ḥaz̤art-i Āyat Allāh al-ʻUẓmá Marʻashī Najafī, 2010), 31–2.

17 For the use of these terms for deadly epidemics in general, and plague in particular, see Dols, *op. cit.* (note 6), 315–6. The terms are used somewhat interchangeably by Fancy and Green, *op. cit.* (note 1).

18

A.D. Lee , War in Late Antiquity: A Social History (Oxford: Blackwell, 2007), 135 . One can learn much about this matter also from more modern military history, as with regard to the American Civil War, the ‘last large-scale conflict before the knowledge of the germ theory of disease. Unsound hygiene, dietary deficiencies and battle wounds set the state for epidemic infection, while inadequate information about disease causation greatly hampered disease prevention, diagnosis, and treatment. Pneumonia, typhoid, diarrhea/dysentery, and malaria were the predominate illnesses.’ Jeffrey S. Sartin, ‘Infectious diseases during the Civil War: the triumph of the “Third Army”’, *Clinical Infectious Diseases*, 16 (1993), 580–4, here from the abstract on p. 580. We are grateful to John Yaphe MD for this reference and for helping us better understand the outbreaks of deadly epidemics at times of widespread crisis and stress.

19 Green, ‘The Four Black Deaths’, (note 1), 1621.

20 Shīrāzī*, Akhbār-i Mughūlān*, 28; Lane (tr.), *The Mongols in Iran*, *op. cit.* (note 14), 52; Rashīd al-Dīn, *Jāmiʿ al-tawārīkh*, ed. Muḥammad Rawshan and Muṣṭafā Mūsawī (Tehran: Nashr-i Alburz, 1373, 1994), II: 989–90; *Rashīduddin Fazlullah’s Jami‘u’t-Tawarikh: A History of the Mongols*, tr. W.M. Thackston (Cambridge MA: Harvard University, Dept. of Near Eastern Languages and Civilizations, 1998–1999), III: 485.

21 Shīrāzī, *Akhbār-i Mughūlān*, 25; Lane (tr.), *The Mongols in Iran*, *op. cit.* (note 14), 51; Rashīd al-Dīn, *Jāmiʿ al-tawārīkh*, II: 989-990; *Rashīduddin Fazlullah’s Jami‘u’t-Tawarikh,* tr. Thackston, III: 485. For these campaigns in general, see John A. Boyle, ‘Dynastic and political history of the Īl-khans’, *The Cambridge History of Iran*, vol. 5: *The Saljuq and Mongol Period*, ed. J.A. Boyle (Cambridge: Cambridge University Press, 1968), 242–5.

22 Gregory Bar Hebraeus (< Bar `Ebrāyā), *Khtobo d’makhtebanuth Zabhne*, published as Paul Bedjan (ed.), *Gregorii Barhebraei Chronicon Syriacum e codd. mss. emendatum ac punctis vocalibus adnotationibusque locupletatum.* (Paris: Maisonneuve, 1890), 506. We are grateful to Raz Amitai-Preiss for this reading and translation. Cf. the translation in Ernest A. Wallis Budge (ed. and tr.), *The Chronography of Gregory Abû ‘l-Faraj, 1225-1286, the Son of Aaron, the Hebrew Physician Commonly Known as Bar Hebraeus …* (London: Oxford University Press, 1932), 431, that was used by Green.

23 Ibn Shaddād al-Ḥalabī, *Taʾrīkh al-malik al-ẓāhir (Die Geschichte des Sultans Baibars)*, ed. A. Ḥuṭayṭ (Wiesbaden: Ludwig Reicher, 1983), 332–3; Muḥyī al-Dīn Ibn ʿAbd al-Ẓāhir, *al-Rawḍ al-ẓāhir fī sīrat al-malik al-ẓāhir*, ed. ʿA-ʿA al-Khuwaytir (Riyad: Muʾassasat Fuʾād, 1976), 87–8; Reuven Amitai-Preiss, *Mongols and Mamluks: The Mamluk- Īlkhānid War, 1260–1281* (Cambridge: Cambridge University Press, 1995), 63.

24 Muḥammad b. Sālim Ibn Wāṣil, *Mufarrij al-kurūb fī akhbār banī ayyūb*, MS. Bibliothèque nationale de France arabe no. 1702, fols. 397b–8a (with the mention of the mice); Ibn ʿAbd al-Ẓāhir, *al-Rawḍ*, *Ibid.*,117–8; Quṭb al-Dīn Mūsā al-Yūnīnī, *Dhayl mirʾāt al-zamān* (Hyderabad: Dāʼirat al-Maʻārif al-ʻUthmāniyya, 1954), I: 498–9; Nāṣir al-Dīn ʿAbd al-Raḥmān Ibn al-Furāt, ed. and tr. M. and U. Lyons and J. Riley-Smith, *Ayyubids, Mamlukes and Crusaders. Selections from the Tārīkh al-Duwal wal-Mulūk of Ibn al-Furāt* (Cambridge: Heffer, 1971), I: 52–3 (tr. II: 43–4; derived clearly from Ibn ʿAbd al-Ẓāhir); Peter Thorau, *The Lion of Egypt: Sultan Baybars I and the Near East in the Thirteenth Century*, tr. P.M. Holt (London: Longman, 1992), 143.

25 Here we can also note that Bar Hebraeus’ own Arabic version of his chronicle does not mention this entire tangent and thus we do not find any mention of ‘severe famine and pestilence’ in the Arabic account. See Ibn al-ʿIbrī, *Taʾrīkh mukhtaṣar al-duwal*, ed. A. Ṣāliḥānī, 2^nd^ edition (Beirut: al-Maṭbaʿa al-Kathūlikiyya, 1958), 272, where he concludes the description of the conquest of Baghdad, and the tangent should begin were it not missing. Bar Hebraeus’ own Arabic version is not to be confused with a modern Arabic translation from the Syriac, which was consulted by Fancy and Green, *op. cit.* (note 1), 160, n. 15.

26 ‘Ibn al-Fuwaṭī’ (but now, Anon.), *al-Ḥawādith al-jāmiʿa wa’l-tajārib al-nāfiʿa*, ed. M. Jawād (Baghdad: al-Maktaba al-ʻArabiyya, 1351 H, 1932–3), 331. This is our translation, slightly different from that of Hend Gilli-Elewy, ‘Al-Ḥawādiṯ al-ğāmiʿa: a contemporary account of the Mongol conquest of Baghdad, 656/1258’, *Arabica*, 58, 5 (2011), 353–71, here 368. See the introduction of that article for this work and the contemporary (or near contemporary) nature of its author, who in high probability was not Ibn al-Fuwaṭī.

27 Shīrāzī*, Akhbār-i Mughūlān*, 34; Lane (tr.), *The Mongols in Iran*, *op. cit.* (note 14), 55.

28 Anon., *Ḥawādith*, 331.

29 We can already note that Bar Hebraeus’ work—neither in the Syriac nor Arabic versions—apparently had little or no impact on the Arabic historiography of the Mamluk Sultanate, although perhaps some historians were familiar with it; on the other hand, the anonymous chronicle attributed to Ibn al-Fuwaṭī clearly was read and used by some Mamluk historians, as seen below.

30 Fancy and Green, *op. cit.* (note 1), 157.

31
*Ibid.*, 159

32 The *Hijri* year of 656 began on 15 January 1258 and thus is more-or-less equal to that Common Era year of 1258.

33 One might also quibble about how these generations were organized. Al-Yūnīnī (b. 1242) was a young contemporary (and observer) of these events.

34 Fancy and Green, *op. cit.* (note 1), 169, refer to Conrad, who concluded that ‘it was only in the mid-thirteenth century that *ṭāʿūn* (plague) came to be widely seen as special type of *wabāʾ* (epidemic)’. Yet, Conrad is explicitly referring here to lexicographers and religious treatises (and writes of ‘the beginning of the fourteenth century’, wondering, indeed, why the terms become almost synonymous nearly half a century before the Black Death); Lawrence I. Conrad, ‘Tā‘ūn and wabā’: conceptions of plague and pestilence in early Islam’, *Journal of the Economic and Social History of the Orient*, 25 (1982), 291. Dols notes that ‘although *ṭāʿūn* may have the generic sense of “an epidemic”, it is used consistently in the late medieval Arabic texts in the specific sense of “a plague”.’ However, he also states that ‘in many cases it is difficult to determine whether an illness is plague without additional corroborative evidence’. Dols, *The Black Death* (note 6), 315–6.

35 On Mamluk (and Ayyubid) historiography, see Donald P. Little, *An Introduction to Mamlūk Historiography: An Analysis of Arabic Annalistic and Biographical Sources for the Reign of al-Malik an-Nāṣir Muḥammad ibn Qalāʾūn* (Wiesbaden: Franz Steiner, 1968); *Ibid.*, ‘Historiography of the Ayyūbid and Mamlūk Epochs’, in Carl Petry (ed.), *The Cambridge History of Egypt*, vol. 1: *Islamic Egypt, 640–1517* (Cambridge: Cambridge University Press, 1998), 412–44; Ulrich Haarmann*, Quellenstudien zur frühen Mamlukenzeit* (Freiburg im Breisgau: Klaus Schwarz Verlag, 1970); Li Guo, *Early Mamluk Syrian Historiography: Al-Yūnīnī’s Dhayl Mirʼāt al-Zamān* (Leiden: Brill, 1998). See now the thoughtful survey in Carl F. Petry, *The Mamluk Sultanate: A History* (Cambridge: Cambridge University Press, 2022), 223–42.

36 Anon., *al-Ḥawādith al-jāmiʿa*, 330–1.

37 Between these two paragraphs are about 10 lines of text, dealing with events in southern Iraq at that time.

38 This is our translation, but it draws on that provided by Gilli-Elewy, ‘Al-Ḥawādiṯ al-ğāmiʿa’ *op. cit.*, (note 26), 367–8.

39 Al-Kutubī (Ṣalāḥ al-Dīn Muḥammad b. Shākir)*, ʿUyūn al-tawārīkh*, ed. A. ʿAbd al-Sattār (Cairo: Dār al-Kutub wa’l-Wathā’iq al-Qawmiyya, 1438/2017), 172. Fancy and Green refer to this author as Ibn Shākir, but al-Kutubī is preferred, as that is how he is invariably known, at least in modern scholarly writing about the Mamluks.

40 Ibn Kathīr (ʿImād al-Dīn Abū al-Fidāʾ Ismā‘īl b. ‘Umar), *al-Bidāya wa’l-niyāha* (rpt. of Cairo: Maṭbaʿat al-Saʿāda, 1932–9), XIII: 202–3.

41 Al-ʿAynī (Badr al-Dīn Maḥmūd b. Aḥmad), ʿ*Iqd al-jumān fī ta*ʾ*rīkh ahl al-zamān*, ed. Muḥammad M. Amīn (Cairo: al-Hay’a al-Miṣriyya al-ʿĀmma li’l-Kuttāb, 1407/1987), I: 174, 176, with more than a full page separating the two passages.

42 See for example, Baybars al-Manṣūrī (d. 1325): ‘Those were killed in Baghdad among the armies and the subjects, the members of the elite and the commoners, more than 1,300,000 souls.’ Baybars al-Manṣurī al-Dawādār (Rukn al-Dīn), *Zubdat al-fikra fī ta’rīkh al-hijra*, ed. D.S. Richards (Beirut: Orient Institut der DMG Beirut, 1998), 38; or Ibn Duqmāq (d. 1407): ‘It is said that those who were killed in Baghdad were about 1,330,000 souls.’ Ibn Duqmāq (Ṣārim al-Dīn Ibrāhīm b. Muḥammad b. Aydamur al-ʿAlā’ī), *Nuzhat al-anām fī ta*ʾ*rīkh al-islām* (Beirut: al-Maktaba al-ʿAṣriyya, 1420/1999), 239; and finally, al-Maqrīzī (d. 1442): ‘Hülegü ordered the counting of the dead, and the number reached around 1,000,000 dead, and because of that, the situation was awful there (*wa-talāshat al-aḥwāl bihā*).’ Al-Maqrīzī (Taqī al-Dīn Aḥmad b. ʿAlī), *Kitāb al-sulūk li-maʿrifat duwal al-mulūk*, ed. Muṣṭafā M. Ziyāda (rpt. of Cairo: Dār al-Kutub, 1934–73), I: 410.

43 Al-Dhahabī (Shams al-Dīn Muḥammad b. Aḥmad), *Taʾrīkh al-islām wa-wafayāt al-mashāhīr wa’l-aʿlām*, ed. ʿUmar ʿAbd al-Salām Tadmūrī (Beirut: Dār al-Kitāb ʿArabī, 1987), LVI: 36: ‘It reached us that Hülegü ordered after this to count the dead, and they reached [the number of] 1,800,000 and a bit more. The most truthful [report] is that they reached 300,000. After that they announced an amnesty, and those who hid appeared, a few among many.’ Ibn Taghrī Birdī (d. 1470), cites appreciably this evidence by al-Dhahabī; Ibn Taghrī Birdī (Jamāl al-Dīn Abū al-Maḥasin Yūsuf), *al-Nujūm al-Zāhira fī mulūk miṣr wa’l-qāhira* (undated rpt. of Cairo: Dār al-Kutub al-Miṣriyya (vols. 1–12) and al-Hayʾa al-Miṣriyya al-ʿĀmma li’l-Kitāb (vols. 12–6), 1930–72), VII: 50.

44 Fancy and Green, *op. cit.* (note 1), 163, note this as well: ‘He is also the only contemporary Ayyubid or Mamluk historian who connects the disease outbreak to the mass killing in Baghdad’.

45 Ibn Wāṣil (Jamāl al-Dīn Muḥammad ibn Sālim), *Mufarrij al-kurūb fī akhbār banī ayyūb*, published as Mohamed Rahim (ed.), *Die Chronik des ibn Wāṣil. Ğamāl ad-Dīn Muḥammad ibn Wāṣil. Mufarrij al-Kurūb fī Aḫbār Banī Ayyūb. Kritische Edition des letzten Teils (648/1248-659/1261) mit Kommentar: Untergang der Ayyūbiden und Beginn der Mamlūkenherrschaft* (Wiesbaden: Harrassowitz, 2010), 159–61.

46 After several pages (p. 178), Ibn Wāṣil returns to the matter of *wabāʾ*, noting the spread of the disease in Syria, especially in Damascus, and the many dead. This passage will be discussed in the next section (Urtext 3).

47 The Arabic word *fasād* may well mean moral corruption and overall disorder, here perhaps brought on by the Mongol conquest of Baghdad. At the same time, the intention of actual physical and human rot caused by the siege and its aftermath cannot be discounted. See below for further discussion.

48 Since the deathrate from this disease was low, this was surely not pneumonic plague. See below for a further discussion.

49 For the expression *al-tāfūn* as a synonym for *ṭāʿūn*, see al-Ṭabarī (Muḥammad b. Jarīr), *Jāmiʿ al-bayān ʿan taʾwīl āy al-Qurʾān* (=*Tafsīr al-Ṭabarī*) (Beirut: Dār al-Fikr, 1408, 1988), IX: 30–1. The matter is succinctly discussed in the Hebrew translation of the Qur’an by the late Uri Rubin for Sura 7:133; see U. Rubin, *The Qur’an: Hebrew Translation from the Arabic, Annotations, Appendices and Index* (Tel Aviv: Tel Aviv University Press, 2005) 132, 317. We are grateful to our colleague Dr. Yosef Witztum for helping to elucidate this matter. For the outbreak of *ṭāʿūn* in Amwās in central Palestine west of Jerusalem, see: Justin K. Stearns, ‘Amwās, plague of’, in *Encyclopaedia of Islam*, THREE, ed. by Kate Fleet et al. First published online in 2016. Consulted online on 29 September 2022 https://doi.org/10.1163/1573-3912_ei3_COM_24915. This author does not discuss the exact medical nature of this plague.

50 Muʿadh b. Jabal, subsequently the commander of the Muslim army in Palestine, who later himself died from this epidemic. See *ibid.*

51 Thus, the earlier unspecified ‘disaster’ that afflicted the Children of Israel was *ṭāʿūn* according to this story.

52
*Maṭʿūn* being the passive participle from the root *ṭ-ʿ-n*, i.e. one affected by *ṭāʿūn.* Fancy and Green *op. cit.* (note 1), 164, translate this as ‘plague-stricken’, really begging the question.

53 Surely, a further play on the root *ṭ-ʿ-n*, also the basis for *ṭāʿūn.*

54 On this ‘enigmatic figure venerated throughout the Islamic world who plays a central role in the traditional Islamic worldview’, see Patrick Franke, ‘Khiḍr’, in *Encyclopaedia of Islam*, THREE, edited by Kate Fleet et al. Consulted online on 27 September 2022 https://doi.org/10.1163/1573-3912_ei3_COM_35534. First published online in 2022.

55 Fancy and Green, *op. cit.* (note 1), 169.

56

Justin Stearns , Infectious Ideas: Contagion in Premodern Islamic and Christian Thought in the Western Mediterranean (Baltimore: John Hopkins University Press, 2011), 71–2. On Galen and miasma: Jacques Jouanna , Greek Medicine from Hippocrates to Galen (Leiden: Brill, 2012), 119–36.

57 Fancy and Green, *op. cit.* (note 1), 163, suggest otherwise.

58 Some parallel information is provided by an Egyptian writer of two generations later, Ibn al-Dawādārī (fl. 1330s), in volume VIII of his chronicle *Kanz al-durar.* In his relatively short account of the conquest of Baghdad, Ibn al-Dawādārī cites a work titled *Taʾrīkh Baghdād*, otherwise unknown, by an author he calls Ibn Wāṣil. This appears to be the same author of *Mufarrij al-kurūb.* In any case, this report does not mention an epidemic in Baghdad itself. However, a few pages on, Ibn al-Dawādārī mentions that al-Nāṣir Dāwūd stayed in Damascus, after the news of the Mongol conquest of Baghdad reached him and there ‘he died of *ṭāʿūn* that was this year in Syria’. This would imply that indeed Ibn al-Dawādārī had Ibn Wāṣil’s *Mufarrij al-kurūb*, in front of him while writing *Kanz al-durar.* Ibn al-Dawādārī (Abū Bakr b. ʿAbdallāh b. Aybak), *Kanz al-durar wa-jāmiʿ al-ghurar*, VIII: *al-Durra al-zakiyya fī akhbār al-dawla al-turkiyya*, ed. Ulrich Haarmann (Cairo: Deutsches Archäologisches Institut Kairo, 1971), 34, 36–7.

59 Al-Dhahabī, *Taʾrīkh al-islām*, LVI: 248–9.

60 Abū al-Fidāʾ (al-Malik al-Muʾayyad Ismāʿīl b. ʿAlī), *al-Mukhtaṣar fī taʾrīkh al-bashar* (Cairo: al-Maṭbaʿa al-Ḥusayniyya al-Miṣriyya, 1325/1907), III: 195, 197.

61 Ibn al-Wardī, (Zayn al-Dīn ʿUmar b. al-Muẓaffar), *Tatimmat al-mukhtaṣar fī akhbār al-bashar*, ed. Aḥmad R. al-Badrāwī (Beirut: Dār al-Maʿrifa, 1389,1970), II: 286–7. For Ibn al-Wardī’s dependence on Ibn Wāṣil, see Little, *Introduction*, 66. Another short version of this account is found in al-Kutubī (d. 1363) (*ʿUyūn al-tawārīkh*, 207); and the historical section of the massive encyclopaedia by al-ʿUmarī (d. 1349), *Masālik al-abṣār.* Al-ʿUmarī (Shihāb al-Dīn Aḥmad b. Yaḥya Ibn Faḍl Allāh), *Masālik al-abṣār fī mamālik al-amṣār*, ed. Kamāl Salmān al-Jubūrī and Mahdī al-Najm (Beirut: Dār al-Kutub al-ʿIlmiyya, 2010), XXVII: 245, 247. Little (*Introduction*, 40) writes that al-ʿUmarī took at least part of the historical information for the historical section of his encyclopedia from al-Dhahabī’s *Kitāb duwal al-islām*, a short summary of his longer *Ta’rīkh al-islām* mostly cited here. While al-Ṣafadī (d. 1366) provides a long entry on al-Nāṣir Dāwūd in his 30-plus volume biographical dictionary, *al-Wāfī bi’l-wafāyāt* (much of the entry devoted to poetry), he gives only a short summary of his death from *ṭāʿūn.* Al-Ṣafadī (Ṣalāḥ al-Dīn Khalīl ibn Aybak), *al-Wāfī bi’l-wafāyāt* (Beirut: al-Maʿhad al-Almānī li’l-Abḥāth al-Sharqiyya and Berlin: Klaus Schwarz Verlag, 2009; rpt. of original 1931–2013 edition of Deutsche Morgenlandische Gesellschaft), vol. XIII, ed. Muḥammad al-Hujayrī, 488.

62 Abū Shāma (Shihāb al-Dīn ʿAbd al-Raḥmān b. Ismāʿīl), *Tarājim rijāl al-qarnayn al-sādis wa’l-sābʿ al-maʿrūf bi’l-dhayl ʿalā al-rawḍatayn*, ed. Muḥammad al-Kawtharī (Cairo: Maktab Nashr al-Thaqāfa al-Islāmiyya, 1947), 200.

63

Michael W. Dols , ‘Plague in early Islamic history’, Journal of the American Oriental Society, 94, 3 (1974), 377 .

64 Fancy and Green, *op. cit.* (note 1), 169.

65
*Ibid.*, 165. Fancy and Green write there that al-Yūnīnī and al-Dhahabī were the ‘two towering figures in early Mamluk historiography’. They were certainly important but no more than their contemporary compatriots al-Jazarī and al-Birzālī, let alone the Egyptians Baybars al-Manṣūrī and al-Nuwayrī.

66 Two terms are actually given here for the word ‘slice’, to make sure the reader knows what is being talked about: *ḥizza ya`nī shaqafa.* The first was apparently a regional expression.

67 Today known in European languages as Tamarind (*Tamarindus indic*), the fruit of which (and maybe other parts of the tree too) has many medicinal qualities. See Leigh Chipman, *The World of Pharmacy and Pharmacist in Mamluk Cairo* (Leiden and Boston: Brill, 2010), passim (the long table stretching almost a hundred pages gives many examples of medicines using Tamar Hindī).

68 Ibn al-Furāt (Nāṣir al-Dīn ʿAbd al-Raḥmān ibn Muḥammad), *Taʾrīkh duwal al-mulūk*, MS Vatican. Ar. 726, fol. 203a-b. We are extremely grateful to our colleague, Dr Iyas Nasser, who very carefully reviewed the reading and translation of this passage, helping us with one especially difficult sentence in particular. We note here that this passage is succinctly summarized by al-Maqrīzī (d. 1442) in his *Kitāb al-sulūk* (I: 410): ‘In [this year] there was much epidemic (*al-wabāʾ*) in Syria, and every day 1200 people died in Aleppo. A large number of the population of Damascus died. The price of a *ratl* of *tamar hindi* reached 60 dirhams’. Invariably, Maqrīzī’s source for these years is Ibn al-Furāt’s chronicle.

69 True, Ibn al-Furāt was a much later historian, but he is distinguished by his frequent incorporation of much earlier sources, often contemporaneous to the events being described. For example, just previously in the same folio (203a), Ibn al- Furāt cites the now lost work of Shāfiʿ b. ʿAlī (d. 1330), *Niẓām al-sulūk fī taʾrīkh al-khulafāʾ wa’l-mulūk.* Also, for his extensive account of the battle of ʿAyn Jālūt in September 1260, he gives a rich assortment of sources, including an eyewitness, as well as passages (with authors named) from other early accounts. See Reuven Amitai-Preiss, ‘ʿAyn Jālūt Revisited’, *Tārīḫ*, 2 (1992), 119–50 (rpt. in John France [ed.], *Medieval Warfare 1000–1300* [Aldershot: Ashgate, 2006]), with some comments about the overall value of this historian.

70 Ibn Wāṣil, *Mufarrij al-kurūb*, ed. Rahim, *op. cit.* (note 45), 178.

71 Gamal el-Din el-Shayyal, ‘Ibn Wāṣil’, in *Encyclopaedia of Islam*, Second Edition, edited by P. Bearman et al., Consulted online on 24 February 2023 https://doi.org/10.1163/1573-3912_islam_SIM_3408

72 Al-Yūnīnī (Quṭb al-Dīn Mūsā b. Muhāmmad al-Baʿlabakkī), *Dhayl ʿalā mirʾāt al-zamān fī taʾrīkh al-aʿyān*, ed. ʿAbbās Hānī al-Jarrākh (Beirut: Dār al-Kutub al-ʿIlmiyya, 1434/2013), I: 128 (cf. Ḥaydarabād edition [1955], I: 91).

73 Not 1100 as found in Fancy and Green, *op. cit.* (note 1), 165.

74 Al-Dhahabī, *Ta’rīkh al-islām*, op. cit. (note 43), LVI: 42. Fancy and Green, *op. cit.* (note 1), 166–7, note that al-Malik al-Ashraf Ismāʿīl, a member of the Rasūlid dynasty in Yemen (d. 1401) followed this passage from al-Dhahabī in his *al-ʿAsjad al-masbūk wa’l-jawhar al-maḥkūk wa-ṭabaqāt al-khulafā’ wa’l-mulūk*, ed. Shākir ʿAbd al-Munʿim (Beirut: Dār al-Turāth al-Islāmī, 1975), I: 645. Unfortunately, we were unable to obtain this book.

75 Fancy and Green, *op. cit.* (note 1), 165, do suggest that al-Yūnīnī and al-Dhahabī heavily relied on Ibn Wāṣil, but as noted above, we have proposed an alternative model of textual transmission.

76 And for that matter, it is also connected to Ibn Wāṣil’s account (Account 2) on the sickness of al-Nāṣir Dāwūd.

77 Kenneth L. Gage and C. Ben Beard, ‘Plague’, in Jonathan Cohen et al. (eds.), *Infectious Diseases*, 4^th^ edn (Elsevier, 2017), cited from https://www.sciencedirect.com/topics/pharmacology-toxicology-and-pharmaceutical-science/pneumonic-plague, accessed on 24 February 2023.

78 The recent outbreaks of COVID in Italy, Spain, New York City, and elsewhere are also a vivid reminder that pneumonic plague is not required for mass death from a respiratory disease.

79 See also in Fancy and Green, *op. cit.* (note 1), 163.

80 Ibn al-Khallikān, Shams al-Dīn Aḥmad b. Muḥammad, *Wafāyāt al-*aʿy*ān wa-anbāʾ al-zamān*, ed. Iḥsan ʿAbbās (Beirut: Dār al-Thaqāfa, 1968–72), II: 338. Fancy and Green, *op. cit.* (note 1), write: ‘Despite contracting the disease himself, the only symptom Ibn Khallikān records is that it was *lethal and killed quickly*’. The emphasis is ours because we have not found that particular information in the Arabic text. All the author says is that the disease was widespread, but nothing about overall mortality.

81 Al-Dhahabī, *Ta’rīkh al-islām*, *op. cit.* (note 43), LVI: 251; al- Ṣafadī, *al-Wāfī biʾl-wafāyāt*, ed. al-Hujayrī, *op. cit.* (note 61), XIV: 236. The latter writer cites Ibn Khallikān elsewhere in this entry, so he was clearly aware of the latter’s biographical notice of Bahāʾ al-Dīn. Ibn Taghrī Birdī (*Nujūm*, *op. cit.* (note 43), VII: 62–3) provides a relatively long death notice of him, but without mention of his dying from *wabāʾ.*

82 Abū Shāma (Shihāb al-Dīn ʿAbd al-Raḥmān ibn Ismāʿīl), *Kitāb tarājim rijāl al-qarnayn al-sādis waʾl-sābʿ al-maʿrūf biʾl-dhayl ʿalā al-rawḍatayn*, ed. Muḥammad al-Kawtharī (Cairo: Maktab Nashr al-Thaqāfa al-Islāmiyya, 1947), 200. N.B. that this is a different edition than that used by Francy and Green. Cf. the translation of this passage by Fancy and Green, *op. cit.* (note 1), 163: ‘And there was a lot of *wabāʾ* in the time of Spring, which was one of the stranger things to happen. People fell ill more than usual and many died.’ Here is the Arabic text in the edition that we used: وقع وباء كثير في زمن الربيع وهو أعجب ما يؤرخ فعمّ الناس المرض وكثر الموت

83 Ibn Khaldūn (Walī al-Dīn ʿAbd al-Raḥmān ibn Muḥammad), *Taʾrīkh Kitāb al-ʿibar wa-dīwān al-mubtadaʾ waʾl-khabar fī ayyām al-ʿarab waʾl-ʿajam waʾl-barbar wa-man ʿaṣarahum min dhawī al-sulṭān al-akbar* (Beirut: Dār al-Kitāb al-Lubnānī, 1956–61), V: 789. Here we might mention that we could find nothing on the outbreak of disease in Baghdad after the Mongol conquest, nor epidemics in Syria and Egypt later that year in the long historical parts of Shihāb al-Din Aḥmad b. ʿAbd al-Wahhāb al-Nuwayrī’s *Nihāyat al-arab fī funūn al-adab* (Cairo: al-Mu*ʾ*assasa al-ʿĀmma li‘l-Taʾlīf wa’l-Tarjama wa’l-Tibāʿ, 1927-97), not in the sections dealing with the end of the ʿAbbasid Caliphate, the Ayyubid dynasty, nor the early Mamluk state.

84 Fancy and Green, *op. cit.* (note 1), abstract. Our conclusions would thus also answer ‘the question why these outbreaks have been overlooked in modern historiography of plague’. *Ibid.*

85 Green, *op. cit.* (note 1), 64.

86 Fancy and Green, *op. cit.* (note 1), 167–9; and see above.

## Appendix

From Ibn al-Furāt, *Taʾrīkh duwal al-mulūk*, MS. Biblioteca Apostolica Vaticana Ar. 726, fol. 203a-b. As is to be expected from manuscripts from the period, elements of the spoken language frequently have entered the text, a register that is referred to as ‘Middle Arabic’. We have left these forms as they appear, but to note the deviation from the standard Literary Arabic (what is called by many Classical Arabic), we have added in square brackets كذا = *sic.* We again take the opportunity to thank our colleague from the Department of Arabic Language and Literature at the Hebrew University, Dr Iyas Nasser, for checking our reading and translation, and helping make sense of one particularly recalcitrant sentence. وقال بعض أهل التأريخ لحق الناس في الشام مرض ووبا (كذا) عقيب أخذ بغداد حتى كان الإنسان لا يصل إلى العطارين إلا بالجهد والمشقة واستغنى العطارين (كذا) ونفدت الأشربة والعقاقير وعزّ الأطبا (كذا) وأهل الحجامة وتعطلت المعايش (كذا) وكسدت البضايع (كذا) وكان الناس على العطارين في دمشق كما يكونوا (كذا) في القحط على الخبازين ودام ذلك الحال أياما وأما أهل حلب فكان الموت فيهم أكثر وورد الخبر^87^ أنه خرج منها في يوم واحد ألف ومائتين (كذا) جنازة قال ومات في دمشق على الطريق خلق كثير وفي المرستانات وعزّ القُرّا (كذا = القُرّاء) والمغسلون وبقي أكثر أهل البلد بالحُمَّى والسُّعال // مَرض واحد وتوفي من الأعيان جماعة وأما من لا يُؤبَه^88^ له فخلق كثير وأبيع البطيخ الأخضر في دمشق في هذه المدة كل حزّة يعني شقفة بدرهم وبلغ كل رطل تمر هندي بستين درهم (كذا) وأما الفراريج (كذا) فكانت لا توجد وإن وُجدت يكون الفروج في قدر العصفور بدرهم ثم صار إلى ثلاثة وأربعة دراهم وأما في حلب فبلغ ثمن كلفروج عشرة دراهم وبالجملة يغني عن التفصيل والله أعلم

